# Supplementary material for: Functional linkage of gene fusions to cancer cell fitness assessed by pharmacological and CRISPR-Cas9 screening
Source: Nat Commun. 2019 May 16;10:2198. doi: 10.1038/s41467-019-09940-1 (PMC6522557; doi:10.1038/s41467-019-09940-1)
Supplement: Supplementary file 1 — Supplementary Information [file 41467_2019_9940_MOESM1_ESM.pdf]

Supplementary Information

Functional linkage of gene fusions to cancer cell fitness assessed by pharmacological and CRISPR/Cas9 screening

By Picco G, et al.

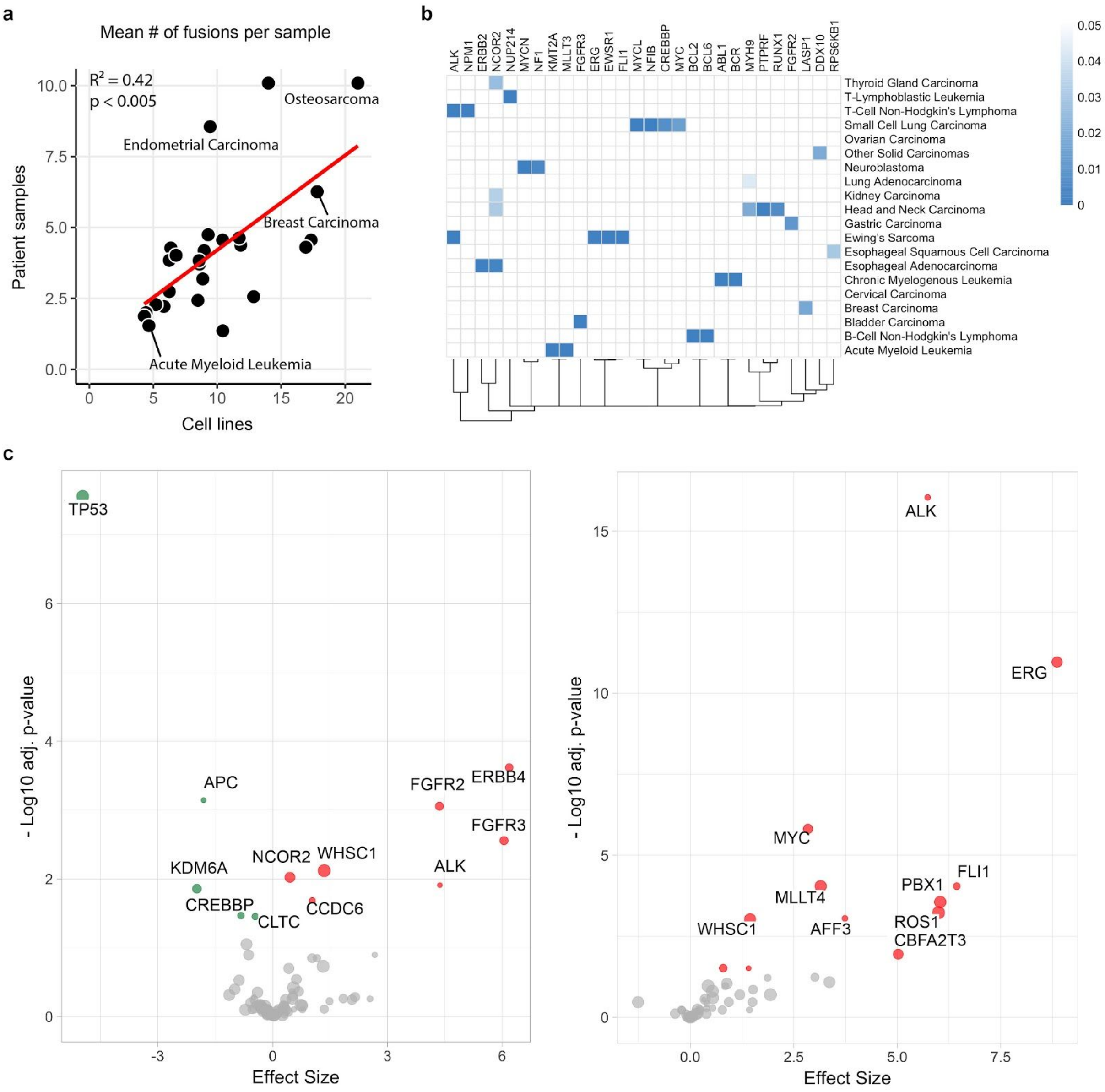

Supplementary Figure 1: Gene fusions detected in cell lines and tumours and their impact on gene expression. (**a**) Mean number of fusion events per sample across different cancer types was significantly correlated between cell lines and patient tumours. Equivalent tissue annotations were present for 27 of 42 cancer types, covering 6,369 fusion events in 704 cell lines and 24,494 fusions events in 5,687 patient samples. P-value computed by Pearson correlation. (**b**) Heatmap for enrichment of fused genes within cancer types. Rows are genes and columns are cancer types. Colour intensity represents the strength of the association (empirical permutation test, adj. p-value). Only interactions with adj. p-value < 0.05 are represented. (**c**) Volcano plots of genes whose expression is significantly altered when at 5-prime end (left panel) and at the 3-prime end (right panel) of fusions. Only cancer

driver genes are represented. Y-axis represents adjusted p-value using Benjamini & Hochberg (FDR) correction, while X-axis represents signed Cohen's d effect size. Circle size is proportional to the number of samples with the gene fused. Colours represent up-regulated (red) and down-regulated (green) genes, respectively. Labels indicate the name of the driver gene.

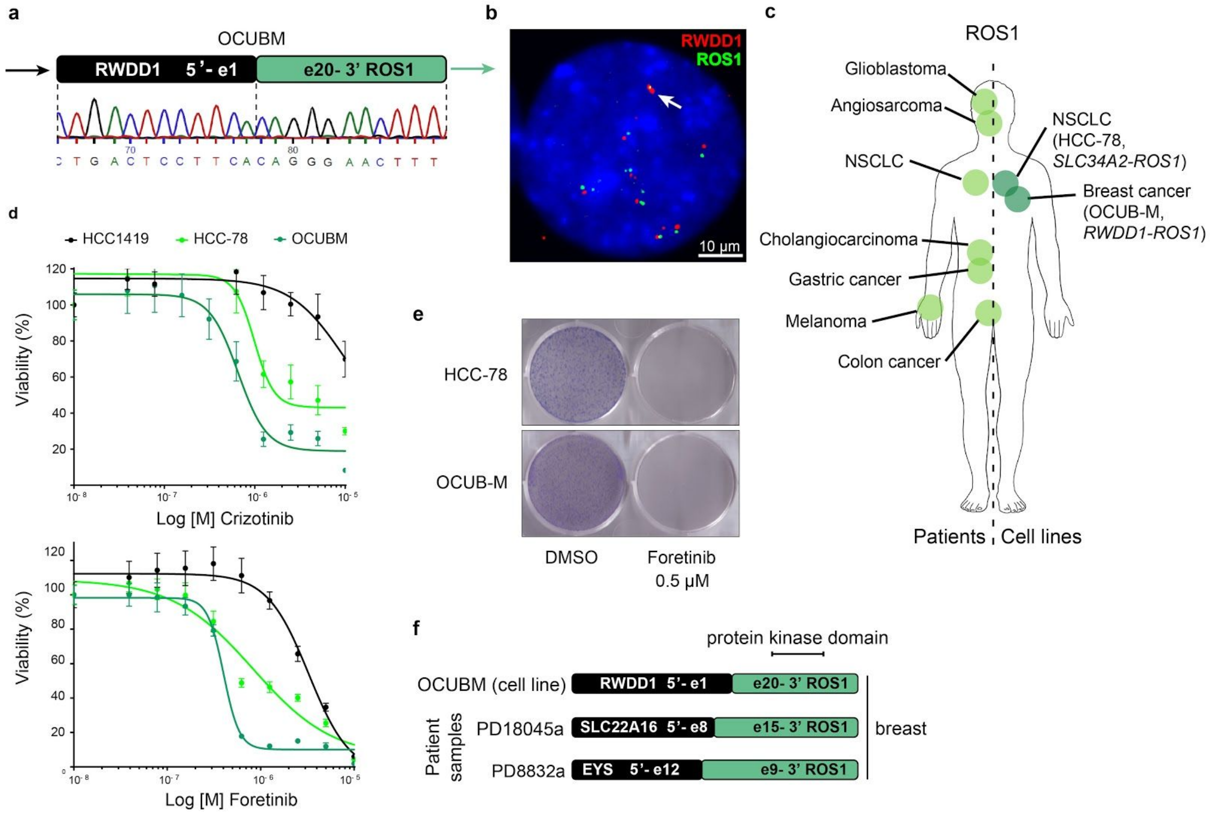

Supplementary Figure 2: Validation of *ROS1* fusion breast cancer cells. (**a**) Sanger sequencing across fusion breakpoint for *RWDD1-ROS1* in OCUBM. (**b**) Interphase FISH image of the *RWDD1-ROS1* fusion in OCUBM cells. (**c**) Oncogenic *ROS1* gene fusions identified in patients previously (left) and cell lines in this study (right). HCC-78, a *ROS1*-rearranged non-small cell lung cancer (NSCLC) used as positive controls for validation experiments is also reported. (**d**) Cell viability assays of OCUBM cells treated with ALK/ROS-inhibitors crizotinib (upper) and foretinib (lower). HCC-78 is a ROS1-rearranged non-small cell lung cancer (NSCLC) and HCC1419 is a fusion-negative control breast carcinoma cell line. Data are expressed as average ± SD of three technical replicates and are representative of two independent experiments. (**e**) Colony formation assays of OCUBM and HCC-78 cells treated with foretinib. Data are representative of three independent experiments. (**f**) Breakpoints of *ROS1*-fusions in breast cancer cell lines and patient samples.

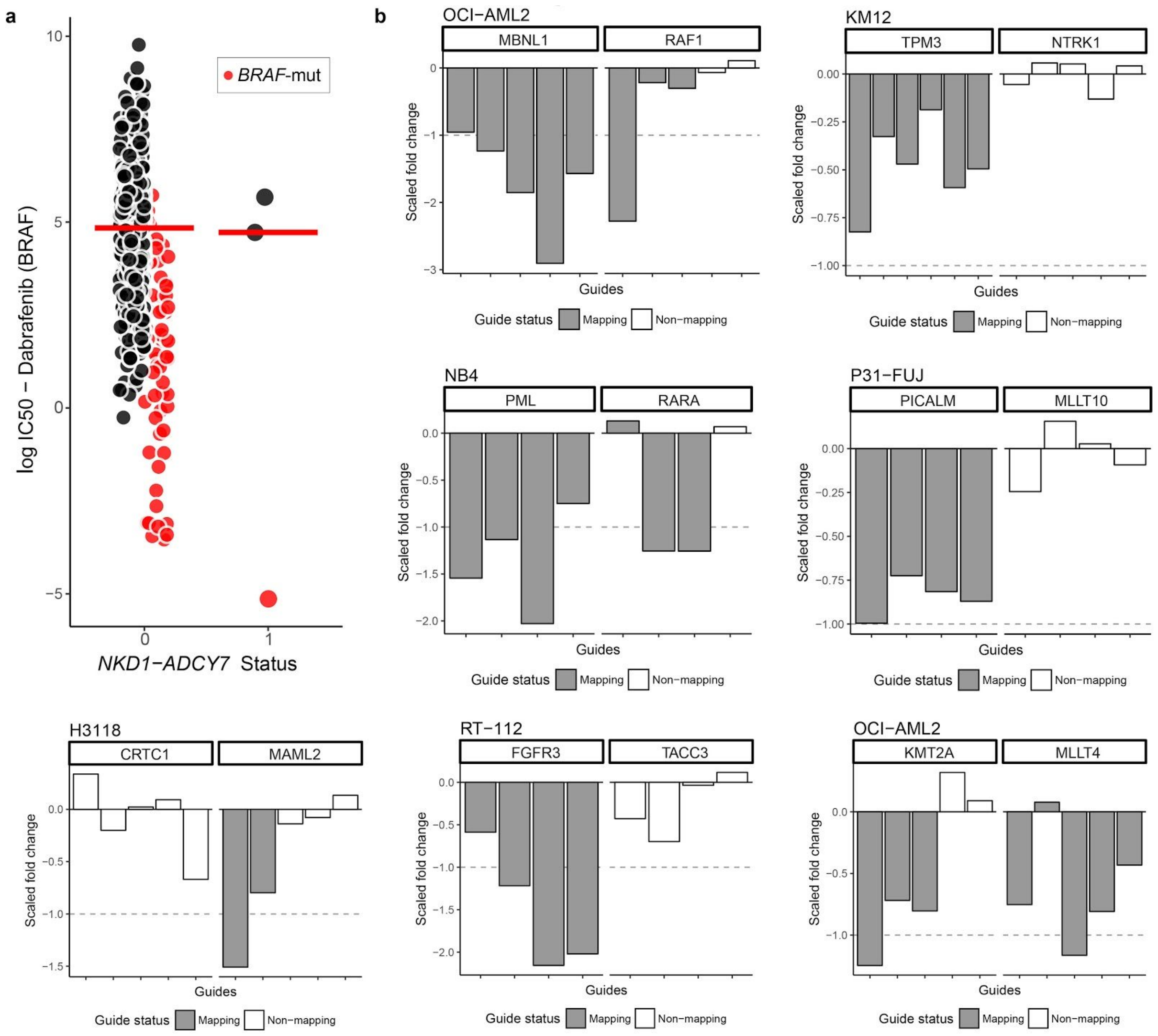

Supplementary Figure 3: Example of a false-positive associations in ANOVA analysis and examples of significant fusion essentiality score for known oncogenic fusions. (**a**) Using cancer events as covariate in our fusion-drug ANOVA successfully excluded false positive associations with fusion events. The association of a *NKD1-ADCY7* fusion with BRAF inhibitor Dabrafenib is confounded by a *BRAF*-mutation in one of the cell lines. This association was no longer significant after implementing the covariates. (**b**) Examples of 7 known oncogenic fusions with a statistically significant fusion essentiality score using CRISPR-Cas9 loss of fitness data.

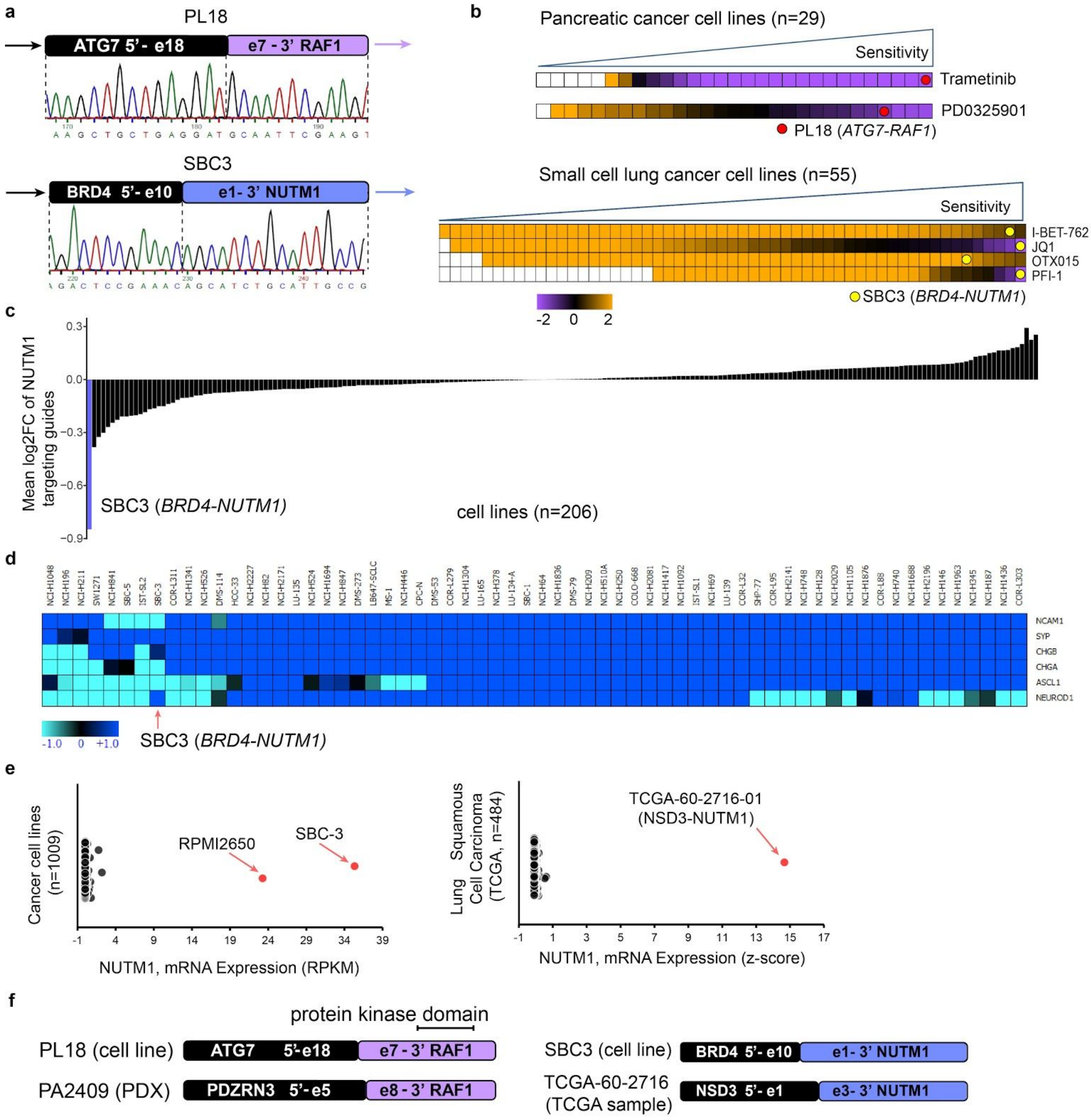

Supplementary Figure 4: Validation of *RAF1* and *NUTM1* rearrangements in pancreatic and small cell lung cancer cells. (**a**) Sanger sequencing across fusion breakpoints for *ATG7-RAF1* in PL18 (pancreatic cell line) and *BRD4-NUTM1* in SBC-3 (small cell lung cancer cell line). (**b**) Heatmaps represents ranked trametinib and PD0325901 log IC50 values in pancreatic cancer cell lines (left) and BET inhibitors log IC50 values in small cell lung cancer cell lines (right) measured by GDSC high-throughput drug screening. PL18 cells are highly sensitive to both MEK inhibitors, SBC-3 cells are highly sensitive to multiple BET inhibitors. (**c**) Across 206 cell lines screened with the Sanger human CRISPR library v1, SBC3 has the highest depletion of *NUTM1* fusion-targeting guides. (**d**) Expression of neuroendocrine markers across 64 small cell lung cancer cell lines. Unlike the majority of

small cell lung cancer cell lines, SBC-3 shows low expression of typical neuroendocrine markers. (**e**) *BRD4-NUTM1* fused cell lines (SBC3 and positive control cell line RPMI2650) show exceptionally high expression of NUTM1 compared to all other cancer cell lines (left panel). We identified a lung squamous cell carcinoma TCGA tumour sample with high NUTM1 expression and a *NSD3-NUTM1* fusion (right panel). Z-score of RNA-seq values for TCGA samples were downloaded from cBioPortal. (**f**) Fusion breakpoints of *RAF1* and *NUTM1* rearrangements in cell lines, PDX models and patient samples. RPKM, Reads Per Kilobase Million.

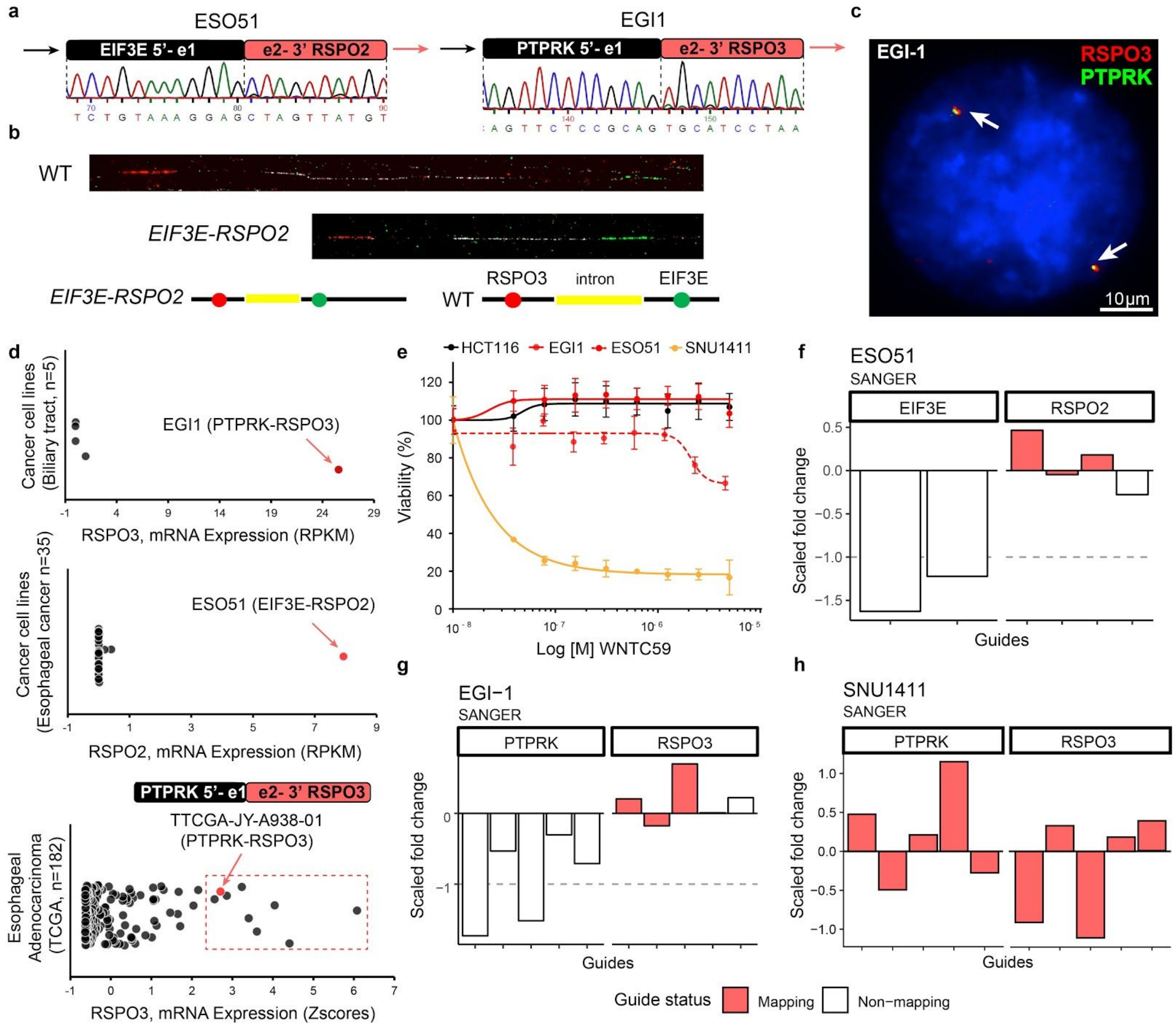

Supplementary Figure 5: Validation of RSPO2/3 rearrangements in oesophageal and biliary tract cancer cells. (**a**) Sequencing across fusion breakpoint for *EIF3E-RSPO2* in ESO51 (an esophageal cancer cell line) and *PTPRK-RSPO3* in EGI1 (a biliary tract cancer cell line). (**b**) Fiber-FISH confirms presence of the *EIF3E-RSPO2* fusion in ESO51. (**c**) FISH shows the presence of the *PTPRK-RSPO3* fusion in EGI-1 (arrows). (**d**) EGI1 and ESO51 are outliers for RSPO2 and RSPO3 expression in esophageal and biliary tract cancer cell lines. We identified an oesophageal cancer patient sample with high RSPO3 expression and a *PTPRK-RSPO3* fusion. Z-score RNAseq values for TCGA samples are from cBioPortal. (**e**) Cell viability assay on EGI1 and ESO51 cells treated with the porcupine inhibitor WNT-C59 for 7 days. Data are expressed as average ± SD of three technical replicates and are representative of two independent experiments. SNU1411 is a positive-control colorectal cancer cell line with a known *PTPRK-RSPO3* fusion. HCT116 is a negative-control colorectal cancer cell line. The RSPO-fusions in ESO51 (**f**), EGI-1 (**g**) and SNU1411

(PTPRK_e13-RSPO3_e2) (**h**) do not confer differential essentiality to mapping versus non-mapping guides in CRISPR screens.

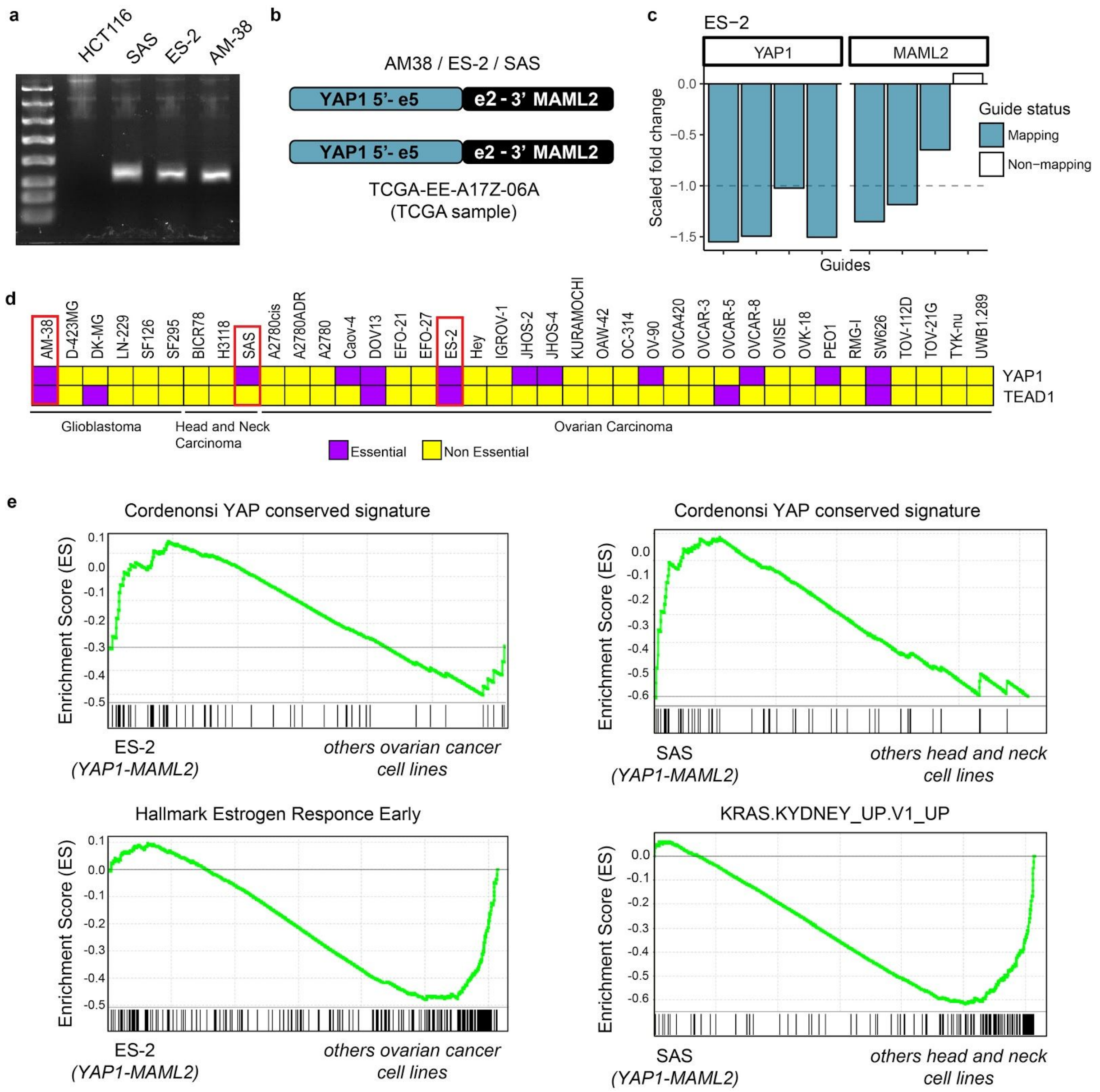

Supplementary Figure 6: *YAP1-MAML2* is a recurrent fusion required for cell fitness. (**a**) PCR validation of the *YAP1-MAML2* fusion in SAS, ES-2 and AM-38. HCT116 is a fusion-negative control. (**b**) The breakpoint of *YAP1-MAML2* is preserved in all three cell lines, as well as in a patient sample identified in TCGA. (**c**) Guides targeting the fused genes are differentially depleted depending on the fusion-mapping status. This holds true across multiple data resources, shown in two independent datasets for ES-2. Broad Institute data shown here and data from Sanger Project Score screen shown in Figure 6. (**d**) Binary essentiality data for *YAP1* and *TEAD1* in ovary, head and neck, and glioblastoma cell lines screened with the Sanger human CRISPR library v1 (n= 38). Cell lines carrying *YAP1* fusions are highlighted in red. (**e**) GSEA of YAP1 expression signature in *YAP1-MAML2* positive ovarian (upper panel, left) and head and neck (upper panel, right) cancer cells vs

fusion negative cell lines of the same tissue type. In the fusion-positive ovarian cancer cell lines ES-2, the gene signature for early estrogen response is downregulated, compared to other ovarian cancer cell lines (left). In the fusion-positive head and neck carcinoma cell line SAS, the KRAS signature is downregulated, with respect to other head and neck carcinoma cell lines (right). The estrogen and the KRAS gene signatures are typical transcriptional hallmarks of the respective cancer types.

Supplementary Table: BAC and fosmid clones used for FISH analysis of fusions.

Cell line	Fusion event	Probe target	BAC and fosmid clones used in the FISH validation
PL18	ATG7-RAF1	ATG7	RP11-177H4
PL18	ATG7-RAF1	RAF1	RP11-275J11
SBC3	BRD4-NUTM1	NUTM1	RP11-194H7
SBC3	BRD4-NUTM1	BRD4	RP11-106J4
EGI1	PTPRK-RSPO3	RSPO3	RP11-193D23
EGI1	PTPRK-RSPO3	RSPO3	WI2-571P5
ESO51	EIF3E-RSPO2	RSPO2	WI2-2136C24
ESO51	EIF3E-RSPO2	RSPO2	WI2-2937O05
ESO51	EIF3E-RSPO2	EIF3E	WI2-0535F14
ESO51	EIF3E-RSPO2	-	RP11-65E1
ESO51	EIF3E-RSPO2	EIF3E	WI2-2809C22
OCUBM	RWDD1-ROS1	RWDD1	WI2-0470A21
OCUBM	RDWW1-ROS1	ROS1	WI2-1624H14
AM-38	YAP1-MAML2	MAML2	G248P8839A4
AM-38	YAP1-MAML2	MAML2	G248P87633H11
AM-38	YAP1-MAML2	YAP1	G248P81419E2
AM-38	YAP1-MAML2	YAP1	G248P84846A9
SAS	YAP1-MAML2	MAML2	G248P8839A4
SAS	YAP1-MAML2	MAML2	G248P87633H11
SAS	YAP1-MAML2	YAP1	G248P81419E2
SAS	YAP1-MAML2	YAP1	G248P84846A9
ES-2	YAP1-MAML2	MAML2	G248P8839A4
ES-2	YAP1-MAML2	MAML2	G248P87633H11
ES-2	YAP1-MAML2	YAP1	G248P81419E2
ES-2	YAP1-MAML2	YAP1	G248P84846A9
