# Supplementary material for: Functional linkage of gene fusions to cancer cell fitness assessed by pharmacological and CRISPR-Cas9 screening
Source: Nat Commun. 2019 May 16;10:2198. doi: 10.1038/s41467-019-09940-1 (PMC6522557; doi:10.1038/s41467-019-09940-1)
Supplement: Supplementary file 3 — Description of Additional Supplementary Files [file 41467_2019_9940_MOESM3_ESM.pdf]

Description of Additional Supplementary Information

File Name: Supplementary Data 1
Description: : Annotation of 1,034 human cancer cell lines used in our study and the source of RNA-seq data. All cell lines are part of the GDSC cancer cell line project (see Cell Model Passport and COSMIC IDs).

File Name: Supplementary Data 2
Description: List and annotation of 10,514 fusion transcripts identified in 1,011 cell lines.

File Name: Supplementary Data 3
Description: Significant results from differential gene expression for recurrent fusions.

File Name: Supplementary Data 4
Description: Annotation of gene fusions for aberrant expression of the 3-prime end gene.

File Name: Supplementary Data 5
Description: High-throughput cell line drug sensitivity data (IC50's) used in this study together with compound annotation. Column names are COSMIC id's of cell lines.

File Name: Supplementary Data 6
Description: Significant ANOVA results from the drug-association analysis using cancer functional events.

File Name: Supplementary Data 7
Description: Significant ANOVA results from the drug-association analysis using gene fusions.

File Name: Supplementary Data 8
Description: Fusion essentiality score and significance calculation for all 2,821 fusion transcripts with mapping guides. For 525 fusion transcripts where multiple data sets contained mapping guides, both are reported.

File Name: Supplementary Data 9
Description: List of primers sequences used to validate gene fusions.
